# Probiotics reduce repeated water avoidance stress-induced colonic microinflammation in Wistar rats in a sex-specific manner

**DOI:** 10.1371/journal.pone.0188992

**Published:** 2017-12-15

**Authors:** Ju Yup Lee, Nayoung Kim, Ryoung Hee Nam, Sung Hwa Sohn, Sun Min Lee, Daeun Choi, Hyuk Yoon, Yong Sung Kim, Hye Seung Lee, Dong Ho Lee

**Affiliations:** 1 Departments of Internal Medicine, Seoul National University Bundang Hospital, Seoungnam, South Korea; 2 Department of Internal Medicine, Keimyung University School of Medicine, Daegu, South Korea; 3 Department of Gastroenterology and Digestive Disease Research Institute, Wonkwang University School of Medicine, Iksan, South Korea; 4 Department of Pathology, Seoul National University Bundang Hospital, Seoungnam, South Korea; University of Nevada School of Medicine, UNITED STATES

## Abstract

The colonic response to stress is greater in female rats than in male rats. The aim of this study was to evaluate the effect of probiotics in the repeated water avoidance stress (rWAS)-induced colonic microinflammation model of Wistar rats in a sex-specific manner. The three groups (no-stress, WAS, and WAS with probiotics) were exposed to r-WAS for 1 h daily for 10 days, and *Lactobacillus farciminis* was administered by oral gavage for 10 days to animals in the probiotics group. The visceromotor response (VMR) to colorectal distension (CRD) was assessed using a barostat and noninvasive manometry before and after WAS exposure. Immunohistochemistry for mast cells and real-time polymerase chain reaction (RT-PCR) for detection of mucosal cytokines were performed using distal colon tissue after the animals were sacrificed. Significant reduction of VMR to CRD (visceral analgesia) was observed at 60 mmHg in the female WAS group (*P* = 0.045), but not in males. In addition, the female WAS with probiotics group showed a significantly lower colonic mucosal mast cell count in comparison to the female WAS group (*P* = 0.013), but this phenomenon was not observed in the male group. The colonic mucosal mRNA levels of interferon-γ (*IFNR*), tumor necrosis factor-α (*TNFA*), interleukin (*IL*) 6, and *IL17* were higher in the female WAS group than in the male WAS group. The mRNA levels of *IFNR*, *TNFA*, and *IL6* were significantly decreased in WAS females who received probiotics (all *P* < 0.050). In conclusion, rWAS is induced in a sex-specific manner. A 10-day-long treatment with *L*. *farciminis* is an effective therapy for rWAS-induced colonic microinflammation in female rates, but not in male rats.

## Introduction

Irritable bowel syndrome (IBS) is a common functional gastrointestinal disorder, and symptoms include chronic abdominal pain and change in bowel habits without structural abnormalities [1]. Stress, increased mucosal permeability, visceral hypersensitivity, and microbiota alteration are thought to be possible causes of IBS [2]. Among them, stress is an important factor in the onset, maintenance, and deterioration of IBS [3]. A recent meta-analysis demonstrated that IBS is more prevalent in females than in males, with the female to male ratio for the odds of IBS being 1.67 [4]. This higher prevalence of IBS in females can be explained by the sex-related differences in stress response, because women are more prone to stress and developing anxiety and depression [5, 6], and have stronger brain response or cognitive dysfunction in response to stress [7]. Although several studies from Asia have reported a higher prevalence of IBS in men than in women, other studies from Asia have reported non-significant differences in prevalence of IBS between men and women [8].

Men and women also show differences in the symptoms and severity of IBS. There is a greater prevalence of symptoms like constipation and bloating in women than in men [9]. Abdominal pain scores for men and women with IBS are similar; however, men report diarrhea whereas women report constipation more often [10, 11]. These differences may be attributed to the sex-related differences in gut microbiota owing to the modulation of gut bacteria by steroidal sex hormones, especially estrogen. A similar effect is observed in the animal model of water avoidance stress (WAS) that mimics human psychological stress [12]. WAS causes symptoms similar to diarrhea-type IBS in rodents, and recent study has shown that WAS increased bowel frequency, mucosal mast cell count, and mucosal interleukin-1β (*IL1β*) levels, especially in female rats [13].

Probiotics can change the composition and balance of the intestinal microbiota in the colonic lumen and mucosal surface. They can inhibit micro-inflammation of the intestine, although this mechanism is unclear [14]. In addition, probiotics were reported to have an antinociceptive effect on stress-induced visceral hypersensitivity in rodents, and are known to increase the expression of opioid and cannabinoid receptors, especially in intestinal epithelial cells [15]. Thus, probiotics can be an effective treatment option for IBS and can alleviate symptoms of IBS. However, clinical results for this treatment differed according to the strain of probiotics used, and different mixtures and dosages of these strains [16]. An additional factor for these variable results could be the different proportion of male and female participants in these studies. Because males and females show differences in the course of IBS, sex-related differences may also influence the response to probiotics in the treatment of IBS, but these differences have not been studied adequately. To use probiotics in the treatment of stress-related disorders like IBS, the effect of sex differences on the response to probiotics must be studied.

Thus, the aim of this study was to evaluate whether the effect of probiotics in a Wistar rat model of rWAS-induced colonic microinflammation is dependent on the sex of the animal, and to investigate the underlying mechanism.

## Materials and methods

### Animals

Male and female Wistar rats (Orient Co., Ltd., Seoul, Korea) were housed in cages (2 animals/cage) maintained at a temperature of 23°C in a 12/12-h light/dark cycle. They were housed under specific pathogen-free conditions, with ad libitum-only Purina rat chow and water, without enrichment [13]. After 1 week of adaptation, 7-week-old male and female Wistar rats weighing 216–282 and 158–202 g, respectively, were used in the experiments. All of the experimental procedures were approved by the Institutional Animal Care and Use Committee (IACUC) of Seoul National University Bundang Hospital (IACUC No. BA1403-149/016-01).

### Experimental protocol

**Fig 1:**
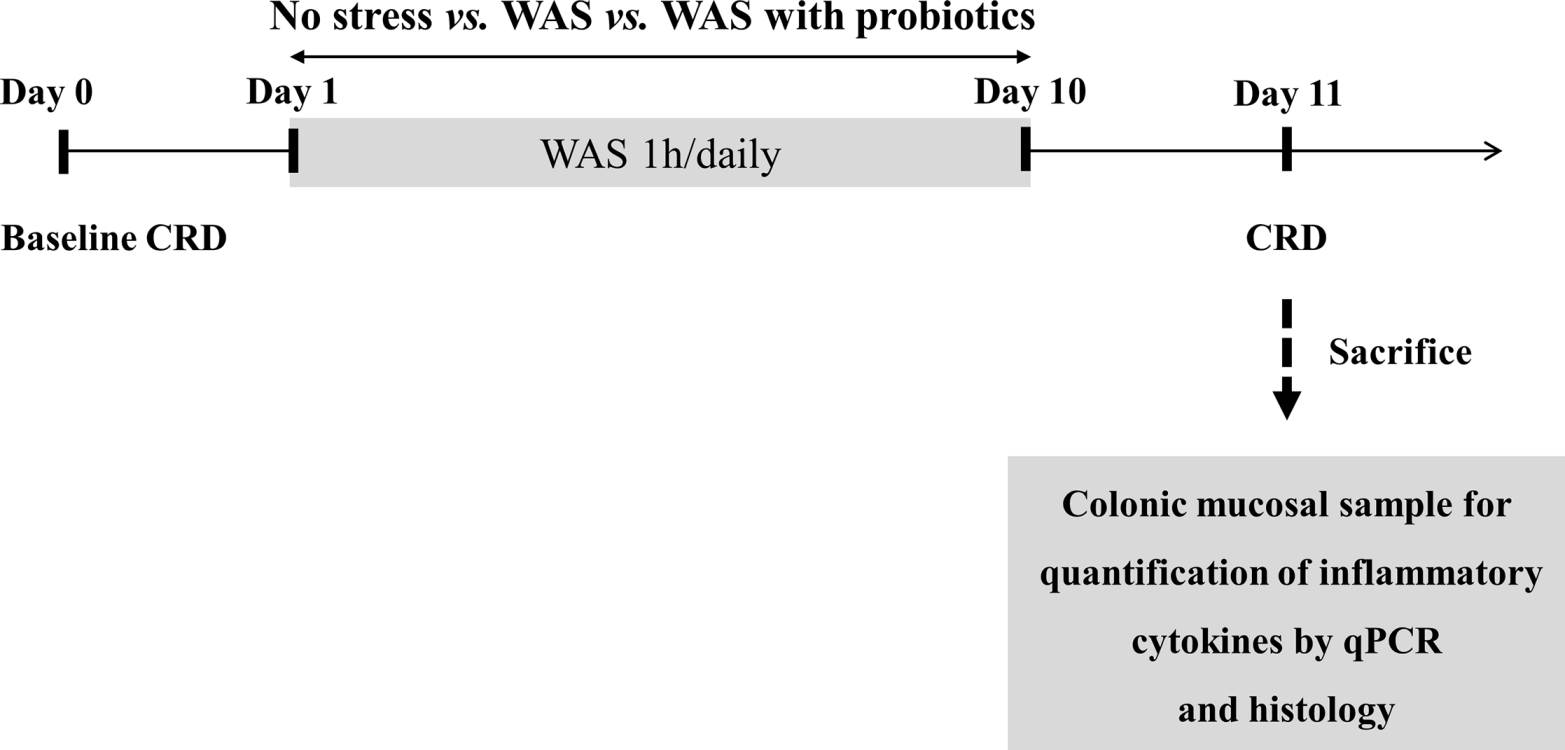
Experimental design. CRD, colorectal distention; WAS, water avoidance stress; qPCR, quantitative polymerase chain reaction.

Healthy Wistar rats of equal age were divided into three groups; no-stress, WAS, and WAS with probiotics groups (Fig 1). There were no inclusion and exclusion criteria for the animal recruitment. During the experiment the case of a sudden decrease in weight due to stress or perforation during colorectal distension did not occur. Regarding the power of the study we did not calculate the adequate sample size mainly because we could not guess how much probiotics affects the WAS-induced microinflammation of colon at this animal model. Animals in the probiotics group were administered a suspension of *Lactobacillus farciminis* (10^11^ CFU/day/rat) using oral gavage for 10 days, whereas the other groups received an oral dose of the vehicle (0.9% NaCl wt/vol in water) for the same period [17]. All the rats in each group were trained in Bollman cages for 60 min/day for 3 days before day 0. The rats were exposed to 1 h of WAS or sham WAS daily for 10 consecutive days one hour after probiotics or oral dose of the vehicle. On day 0 and day 11 (24 h after the last WAS session), the rats were subjected to the CRD protocol and VMR was measured. Immediately after the last CRD session on day 11, all rats were sacrificed by CO_2_ inhalation, and blood and distal colon samples were collected (Fig 1).

### Repeated water avoidance stress

The rWAS was applied as described in previous reports [18]. We used platforms of different sizes for male and female rats to avoid variation in the stress response associated with more comfortable postures because female rats weigh less than male rats. In other words, depending on sex, each Wistar rat was placed on a different glass platform (5.8-cm length × 5.8-cm width × 6.0-cm height for male rats and 5.0-cm length × 5.0-cm width × 6.0-cm height for female rats), which had been fixed to the center of a standard plastic cage (26.7-cm length × 48.3-cm width × 20.3-cm height) filled with warm water (25°C) to 1 cm below the height of the platform, for 1 h for 10 consecutive days between 8 and 10 a.m. [13]. The rats in the no stress group were placed on the same platform, which was now attached to a container not filled with water, for 1 h. All the rats were kept in pairs in their home cage and placed individually in their WAS cage.

### Measurement of fecal pellet output

The total number of fecal pellets expelled by each rat was counted at the end of each 1-hour session of rWAS.

### Assessment of visceral pain response to colorectal distension

This visceral pain response to CRD was assessed using the noninvasive manometric method that Larauche et al. [18] recently developed and validated for use in mice and rats. Briefly, a PE 50 catheter was taped 3.5 cm below the pressure sensor of a miniature pressure transducer catheter (SPR-524 Mikro-Tip catheter; Millar Instruments, Houston, TX, USA). A custom-made balloon (2 cm wide × 5 cm long) made of an infinitely compliant polyethylene plastic bag was tied over the catheter at 1 cm below the pressure sensor with a silk suture [13]. The rats were anesthetized with isoflurane (3% in O_2_), and a lubricated balloon-pressure sensor catheter was inserted into the rectum and distal colon so that the distal end of the balloon was positioned 1 cm from the anus, and the catheter was secured to the tail using surgical tape. Each animal was placed in a Bollman cage covered with a blanket and rested for 30 min before the CRD. Each balloon was connected to the barostat (Distender Series IIR Dual Drive Barostat; G & J Electronics Inc., Toronto, Canada), and a pressure sensor was connected to the preamplifier (PCU-2000; Millar Instruments, Houston, TX, USA) [13]. The CRD protocol consisted of two 60-mmHg CRD sessions in which the balloon was unfolded, immediately followed by two series of graded phasic distensions to constant pressures of 10, 20, 40, and 60 mmHg. Each CRD lasted 20 s and was applied at 4-minute interstimulus intervals. Data analysis was performed using the method described in a previous study [18, 19]. The intracolonic pressure (ICP) signal was obtained using MATLAB software (R2014b; The MathWorks, Natick, MA, USA), and the analysis protocol was programmed by W. Jo [13]. The visceromotor reflex (VMR) was defined as the increase in the area under the curve of pICP during CRD over the mean values of the pre- and post-distension 20-s periods and was quantified using the MATLAB software. Each CRD pressure was repeated twice, and the pre-, intra-, and post-CRD values were averaged for each pressure [13].

### Assessment of colonic mucosal mast cells

A 1-cm-long portion of the distal colon was obtained from each rat and fixed in 10% buffered formalin for histological analysis [13]. The specimens were embedded in paraffin, sectioned perpendicularly to the lumen (section thickness, 4 μm), and mounted on a slide glass [13]. The immunohistochemistry (IHC) staining was performed using an automated immunostainer (BenchMark XT; Ventana Medical Systems, Tucson, AZ, USA) according to the manufacturer’s instructions. The slides were incubated with primary antibodies and a mouse monoclonal anti-mast cell tryptase antibody (Abcam Inc., Cambridge, MA, USA). The negative IHC control was incubated in a solution not containing the primary antibody. Sections were then counterstained with hematoxylin for 4 min to stain the nucleus and then dehydrated, cleared, and mounted in synthetic mountant. Photographs of tryptase positive cells were obtained from 6 to 8 non-overlapping areas on two immunostained slides per rat under a light microscope (Carl Zeiss, Jena, Germany) linked to a computer-assisted image analysis system. The number of cells stained with the primary antibody was counted in all the photographs by three researchers blinded to the animal groupings, and the cell numbers were expressed as mast cells visible per each high-power field (number of cells/hpf).

### Quantification of inflammatory cytokines by quantitative polymerase chain reaction (PCR)

mRNA was isolated from colon tissue using Trizol reagent (Invitrogen, Carlsbad, CA) according to the manufacturer’s instructions, and mRNA was quantified using NanoDrop (ND-1000; Thermo Scientific, Wilmington, DE). Complementary DNA (cDNA) was synthesized using the High Capacity cDNA reverse Transcription Kit (Applied Biosystems, Foster City, CA). Real-time quantitative PCR (qPCR) was performed using SYBR Green I Master mix and an ABI Viia7 instrument. The transcript levels of β-actin were used for sample normalization. The sequences of the rat primers were as follows: *IL1B* (FW 5′-GCA TCC AGC TTC AAA TCT CA-3′; RW 5′-ATC ATC CCA CGA GTC ACA GA-3′), interferon-γ (*IFNR*) (FW 5′-CGA ATC GCA CCT GAT CAC TA-3′; RW 5′-GAC TCC TTT TCC GCT TCC TT-3′), tumor necrosis factor-α (*TNFA*) (FW 5′-GCC GAT TTG CCA TTT CAT AC-3′; RW 5′-TGG AAG ACT CCT CCC AGG TA-3′), *IL6* (FW 5′-CCG GAG AGG AGA CTT CAC AG-3′; RW 5′-CAG AAT TGC CAT TGC AAC AAC-3′), *IL17* (FW 5′-GTG AAG GCA GCG GTA CTC A-3′; RW 5′-TTC TGG AGC TCG CTT TTG A-3′), *PRSS1* (FW 5′-CCA AGT GAG ACT GGG AGA GC-3′; RW 5′-GTT GGG GTG CTT GAT GAT CT-3′), *PRSS2* (FW 5′-CCA AGT GAG ACT GGG AGA GC-3′; RW 5′-TCC TAT CGA AGT TGG GAT GC-3′), *PRSS3* (FW 5′-AGC CGC TCA CTG CTA CAA AT-3′; RW 5′-AAT TGC TCA CCA CCC TCA AC-3′), and Rat/Mouse *ACTB* (FW 5′-CCA GAG CAA GAG AGG TAT CC-3′; RW 5′-CTG TGG TGG TGA AGC TGT AG-3′).

### Statistical analyses

Data are expressed as means ± stand error of means (SEM). Sex-related differences in VMR to CRD over time between the groups were analyzed using one-way ANOVA or two-way ANOVA followed by the Bonferroni post hoc test. Continuous and categorical variables were compared among the groups (no-stress, WAS, and WAS with probiotics) by using the Kruskal-Wallis and Fisher’s exact tests, respectively. *P* values of < 0.05 were considered statistically significant. All the statistical analyses were performed by using the SPSS version 20.0 software (SPSS Inc., Chicago, IL, USA).

## Results

### Fecal pellet output during water avoidance stress

**Fig 2:**
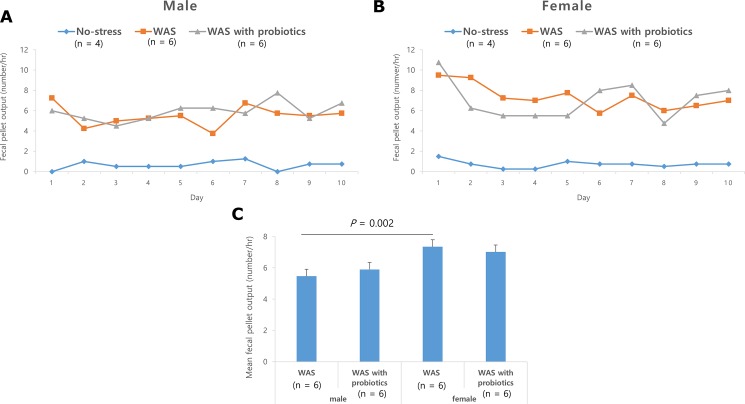
Fecal pellet output (FPO) induced by repeated water avoidance stress (WAS). (A) Daily FPO for 10 days in male with WAS group regardless of probiotics were significantly higher than in control.(B) This phenomena were similar in female.(C) The mean FPO was higher on female WAS group than male WA group (*P* = 0.002). Data are expressed as means ± SEM.

The FPO of the WAS and WAS with probiotics groups was significantly higher than that of the no-stress group in both male and female rats (Fig 2A and 2B), but there was no significant difference in the FPO between the WAS and WAS with probiotics groups, in both males and females (Fig 2C). Within the WAS group, the mean FPO in females was significantly higher than that of the males (5.5 ± 0.3 vs 7.4 ± 0.4/h; *P* = 0.002) (Fig 2C).

### Sex-related difference in VMR to CRD

**Fig 3:**
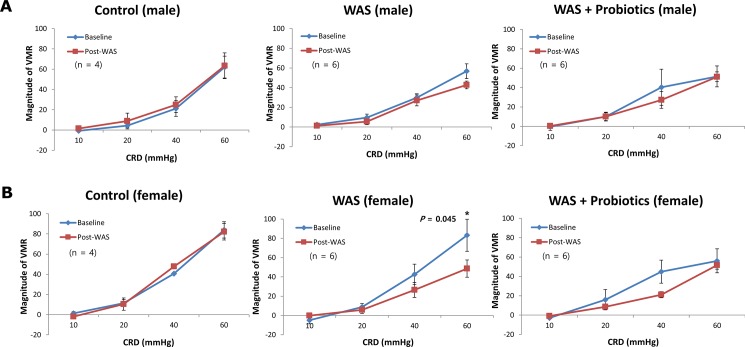
Visceral analgesic responses induced by repeated water avoidance stress (WAS). (A) There was no difference of visceromotor response (VMR) to colorectal distension (CRD) before and after WAS regardless of treatment of probiotics in male. (B) In contrast VMR to CRD (visceral analgesia) was decreased at 60 mmHg (*P* = 0.045) in comparison to baseline in female rats. This WAS-associated visceral analgesia disappeared treatment of probiotics.

In male rats of the no-stress, WAS, and WAS with probiotics group, no statistically significant changes in VMR due to any CRD pressure were observed between baseline (day 0) to day 11 (Fig 3A). In female rats of the WAS group, the VMR to CRD (visceral analgesia) observed at 60 mmHg was lower than that observed at the baseline (*P* = 0.045). However, after administration of probiotics, this difference disappeared (Fig 3B). Meanwhile, there was no significant difference observed in the VMR to CRD in male rats regardless of the probiotics treatment.

### Sex-related difference in mucosal mast cell count in the distal colon

**Fig 4:**
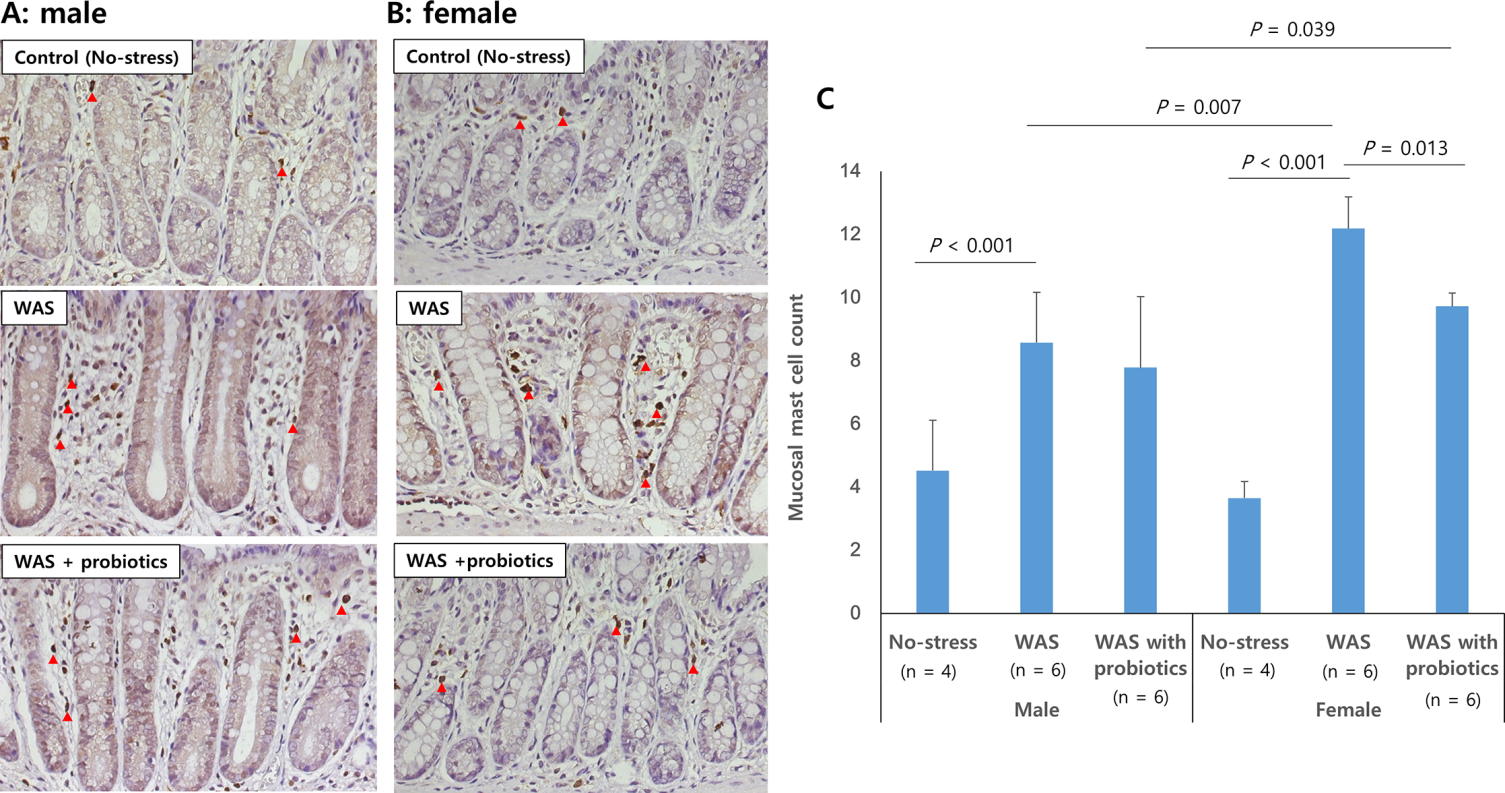
**Number of mucosal mast cells detected after anti-mast cell tryptase antibody staining** (A: male, B: female, red arrow, × 400). Mucosal mast cell counts in distal colon were higher in the water avoidance stress (WAS) group than in the no-stress group in both male and female rats. Female WAS with probiotics group showed significantly lower mast cell count than female WAS group (*P* = 0.013) (C).Data are expressed as means ± SEM.

Mucosal mast cell counts were significantly higher in the WAS and WAS with probiotics group than in the control group in both male and female rats (*P* < 0.001, respectively) (Fig 4). In addition, the mucosal mast cell counts of the female rats of the WAS group were significantly higher than those of the male rats of the WAS group (12.2 ± 1.0/unit area vs. 8.6 ± 1.6/unit area; *P* = 0.007), showing a sex-specific difference. The mucosal mast cell counts in the distal colon of the female WAS with probiotics group were significantly lower than those of the female WAS group (9.7 ± 0.4/unit area vs. 12.2 ± 1.0/unit area; *P* = 0.013); however, there was no significant difference between the male WAS group and male WAS with probiotics group (Fig 4C).

### Mucosal cytokine mRNA levels in the distal colon

**Fig 5:**
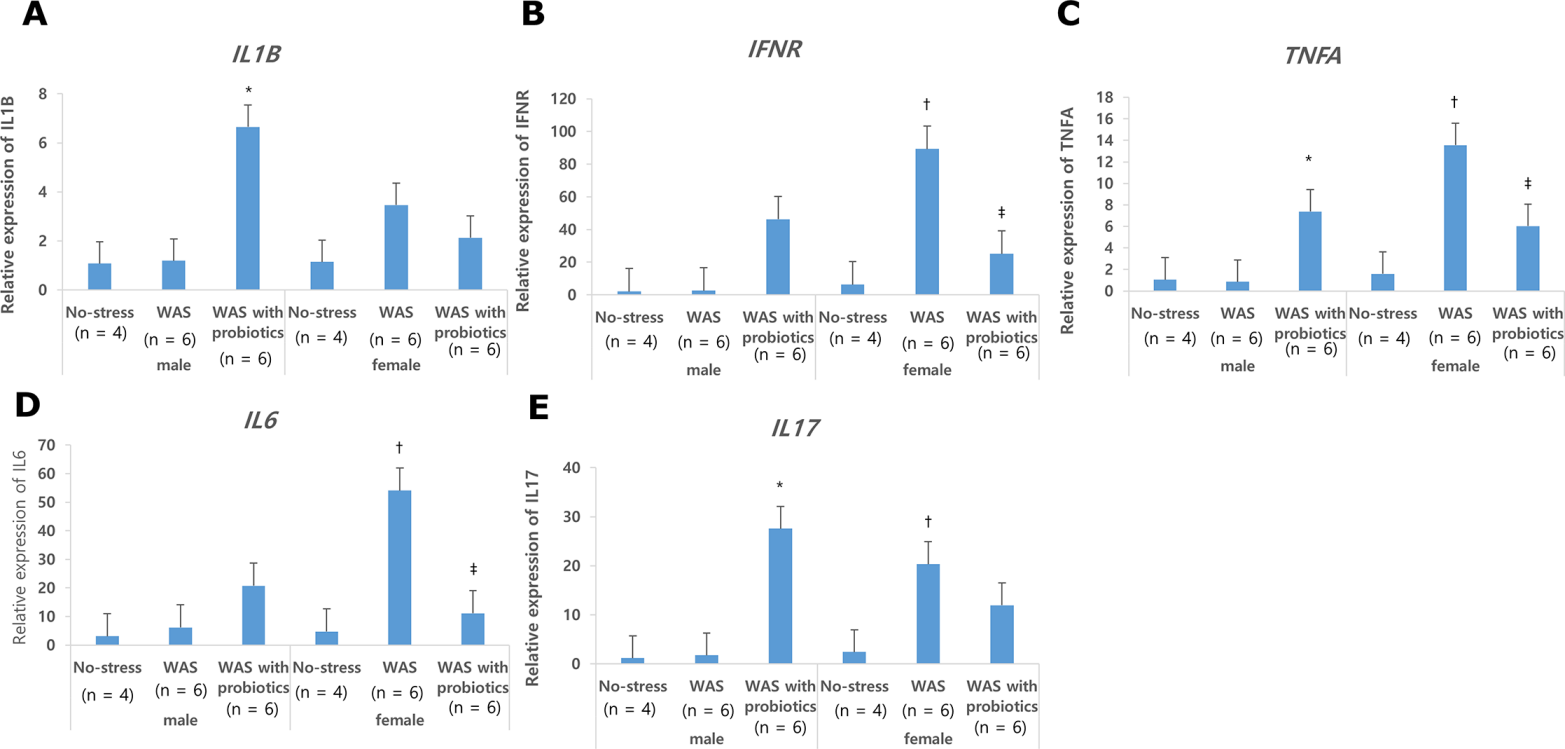
Colonic mucosal mRNA expressions. (A) Mucosal qPCR expression of interleukin-1β (*IL1B*) in distal colon was significantly higher in male rats of the water avoidance stress (WAS) group with probiotics group than in those of the male WAS group. (B, C, D)Mucosal interferon-γ (*IFNR*), tumor necrosis factor-α (*TNFA*), *IL6* expression in distal colon was significantly higher in female rats of the WAS groups than in those of the male rats and significantly decreased only in female rats of the WAS with probiotics group. (E)Mucosal qPCR expression of *IL17* in distal colon was significantly higher in male rats of the WAS group with probiotics group than in those of the male WAS group. Data are expressed as means ± SEM. *P <0.05 compared with no-stress and WAS group in male; ^†^*P* <0.05 compared with female no-stress group; ^‡^*P* <0.05 compared with female WAS group.

**Fig 6:**
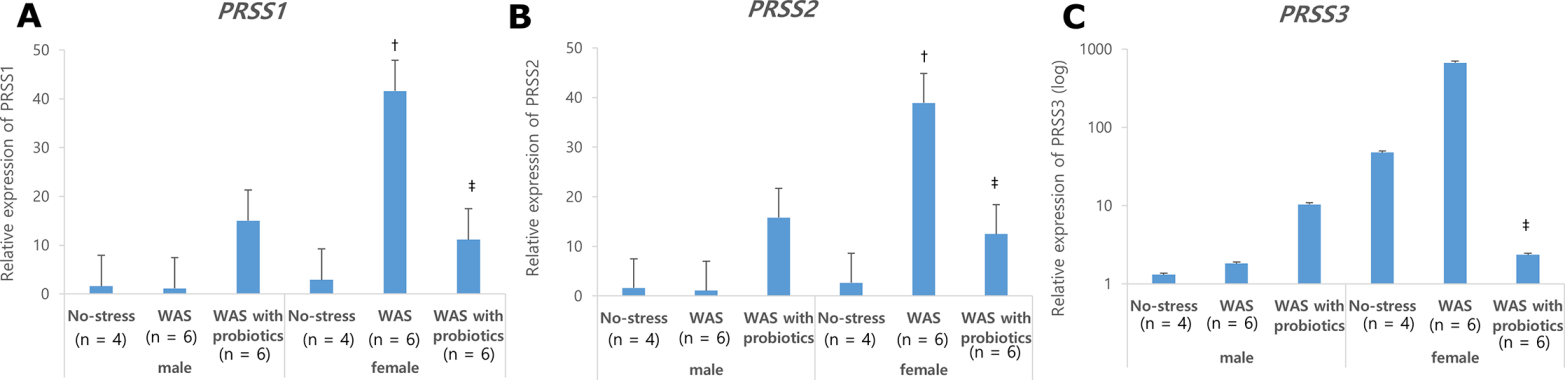
The qPCR expression of *PRSS1*, *PRSS2* and *PRSS3*. **2** (A) The expression of *PRSS1* was significantly higher in female water avoidance stress (WAS) group than those in male WAS group. The treatment of probiotics significantly decreased WAS-induced higher expression of *PRSS1* in female. However, there was no significant change in male. (B) The expression of *PRSS2* was also similar to *PRSS1*in female group. (C) In case of *PRSS3* the treatment of probiotics significantly decreased WAS-induced higher expression of *PRSS1* in female but there was no significant difference between control group and WAS group in both of female and male. *P <0.05 compared with no-stress and WAS group in male; ^†^*P* <0.05 compared with female no-stress group; ^‡^*P* <0.05 compared with female WAS group.

Mucosal mRNA expressions of *IL1B*, *TNFA*, and *IL17* in the distal colon were significantly higher in the male WAS with probiotics group than in the male WAS group and male control group. However, mucosal expressions of *INFR* and *IL6* were not statistically different among the male groups (Fig 5). Mucosal *INFR*, *TNFA*, *IL6*, and *IL17* expressions in the distal colon were significantly higher in the female rats of the WAS group than in the male WAS group (*P* < 0.05) (Fig 6). Female rats of the WAS with probiotics group showed significantly lower mucosal *INFR*, *TNFA*, and *IL6* expressions than the female WAS group (*P* < 0.05) (Fig 6). However, in terms of treatment effect of probiotics on *IL1B* and *IL17*, there was no statistical difference among the female groups (Fig 6). Similarly, there was no statistical difference in mucosal serine protease gene (*PRSS*) expression in the distal colon due to treatment of probiotics in both the male and female groups. The mRNA expressions of *PRSS1* and *PRSS2* were significantly higher in the female WAS group than in the male WAS group (Fig 6) The mRNA expressions of *PRSS1*, *PRSS2*, and *PRSS3* were significantly lower in the female WAS probiotic group, but this effect was not found in the male groups (Fig 6).

## Discussion

In the present study, rWAS (chronic psychological stress) significantly increased the FPO reflecting an increase in bowel frequency, and it induced visceral analgesia in female rats. In addition, mucosal mast cell counts in the distal colon were significantly higher in the female rats of the WAS group, and the modulation effect of probiotics was more prominent in the female rats. Similarly, rWAS provoked mRNA expressions of various colonic mucosal cytokines, and treatment with probiotics decreased these only in the female rats, suggesting that there is a sex-related difference in colonic microinflammation. This implies that the effect of probiotics could be different depending on sex.

Our data demonstrate that female rats are more prone to WAS. Many human studies also revealed that women are more prone to stress and anxiety and therefore are more susceptible to exacerbation of IBS symptoms under stress [5, 6, 20]. The reason of this sex-related difference in IBS is not well understood. However, a previous study suggested that ovarian and stress hormones may be associated with the female predominance of IBS [21]. Sex hormones play a crucial role in the regulation of the brain-gut-microbiota axis, which is involved in IBS pathophysiology. In particular, estrogen has both analgesic or hyperalgesic and pro- or anti-inflammatory effects [7]. These dual effects are thought to affect the female predominance of IBS through a complex mechanism.

Intestinal mast cells are thought to be involved in the pathophysiology of functional gastrointestinal disorders, including IBS [22]. The mast cells are anatomically functional and closely related to the intrinsic and extrinsic nerves of the gastrointestinal tract, intestinal smooth muscles, and secretory glands [23]. In addition, it is believed IBS occurs because of the collapse of brain-gut axis regulation due to early-life stress, and psychiatric comorbidity stimulates intestinal low-grade inflammation and mast cell infiltration [24]. Psychological stress is known to be closely associated with the onset and deterioration of IBS, and this association is greater in females. Exposure to acute or chronic stress affects bowel function and mast cell activation [25]. There have been many studies on contribution of mast cells to stress-induced gut dysfunction [25, 26]. Mast cells secrete tryptase, *TNFA*, and histamine, which induce intestinal barrier disruption and visceral hypersensitivity [22]. In concordance with the results of our previous study [13], we reaffirm that WAS significantly increased the mucosal mast cell count in rats, and this increase was more prominent in the female rats than in the male rats in the present study. Whether mast cell secretion is more prominent in females after psychological stress is not yet known. Estrogen, a female hormone, has been reported to induce mast cell degranulation [27]. In a recent study, Mackey et al. reported that in female mice undergoing restraint stress, intestinal permeability is increased, and the serum histamine secretion is more than in male mice [28]. Therefore, it is presumed that WAS also increases mast cell degradation and secretion. However, we did not measure degranulation of mast cells, and this should be investigated in future studies.

In addition to mast cells, low grade inflammation in IBS activates the hypothalamic-pituitary-adrenal (HPA) axis [29], leading to elevations of inflammatory cytokines, such as *IL6*, *IL1β*, and *TNFA* [30]. These cytokines synergistically stimulate the HPA axis via activation of nociceptive, visceral, and somatosensory afferents [31]. These proinflammatory cytokines markedly released in IBS-diarrhea (IBS-D) patients and may be associated with patient symptoms and anxiety. *IL6* and *IL1β* are also known to increase intestinal tight junction permeability [32]. In addition, *TNFA* is known to induce apoptosis and inflammatory response in intestinal epithelial cells, and it impairs the intestinal tight junction barrier [32]. Current data show that increased levels of serum pro-inflammatory cytokines, such as *IL1β*, *TNFA*, and *IL8*, were higher in IBS patients than in healthy controls [29, 33]. A systemic review and meta-analysis also demonstrated the sex-related differences in inflammatory cytokines in IBS patients. Serum *TNFA* levels were higher in females with IBS than in males with IBS, and *IL10* levels were significantly lower in males than in females with IBS [34]. These sex differences are thought to be due to differences in gut immunity, and individualized treatment based on these differences should be considered. In the present study, expression of *INFR*, *TNFA*, *IL6*, and *IL17* as estimated by qPCR were significantly elevated in female rats compared to male rats. Although information about *IL17* is limited, it is known to be produced in Th17 cells which potently induce tissue inflammation [35]. Since these inflammatory cytokines have complex interrelationships with each other, further studies are needed to demonstrate sex-specific cytokines associated with IBS.

Probiotics have several positive effects in the gut through various actions; reinforcement of mucosal barrier, reduction of permeability, and regulation of the inflammatory response by modulating cytokines and immune reaction. Larauche et al. [19] demonstrated visceral analgesia for stress using a water avoidance stress rat model and demonstrated that this analgesia was further enhanced in the prebiotic enzyme-treated rice fiber diet compared to the standard diet. In the present study, visceral analgesia using WAS was observed in female rats, but the effect of probiotics was not clear, probably owing to differences in the prebiotics and probiotics used and duration of administration. However, we demonstrated that *L*. *farciminis* significantly decreased expression of inflammatory cytokine, such as *INFR*, *TNFA*, *IL6*, and *IL17*, in the distal colonic mucosa in females, which suggests that probiotics have an anti-inflammatory effect, especially in females. However, some inflammatory cytokines, such as *IL1β* and *IL17* were significantly higher in the male WAS with probiotics group, and this suggests that probiotics may have different mechanisms of action between males and females. A similar phenomenon also occurs in the mRNA expressions of the serine protease (*PRSS*) gene. *PRSS* is a trypsinogen-encoding gene that is known to be associated with “trypsin-like activity,” and the PRSS protein is released from IBS patients. Recent studies have shown that PRSS enhances intestinal epithelial permeability and acts on submucosal neurons to cause visceral hypersensitivity [36]. Little is known about the role of probiotics in male and female IBS patients and more research is needed to understand these effects. In one study of the RNA profile of jejunum and ileum after administration of *Lactobacillus reuteri* to mice, *L*. *reuteri* inhibited TNF-α mRNA in jejunum and ileum of male mice, but not in female mice [37]. In another study, fermented milk supplemented with synbiotics altered the nutritional index of the rats and these changes were more prominent in female rats [38].

In the present study, we selected *L*. *farciminis* from numerous strains of known probiotics. This probiotic strain has strong evidence for being effective in the treatment of IBS [17, 39]. It was suggested that administration of *L*. *farciminis* in animal models may prevent WAS-induced epithelial barrier impairment and attenuate visceral hypersensitivity [17, 39]. This preventive effect on epithelial barrier disruption has been observed in an acute stress model [40]. In addition, *L*. *farciminis* showed anti-inflammatory properties in experimental colitis models [41]. The results of the present study also support these reports. Single probiotics alone might not change the entire composition of gut microbiota. Zareie et al. [42] demonstrated that single probiotics (*L*. *farciminis*) can prevent chronic stress induced intestinal abnormalities such as intestinal permeability. One more presumed mechanism is that *lactobacillus* is relatively more affected by WAS and decreases. Watanabe et al. also showed decreased lactobacillus species after WAS [43]. We believe that our data provides evidence for the efficacy of a small number of single-strain probiotics in the treatment of IBS.

Moayyedi et al. [44] analyzed 18 randomized controlled trials (RCTs) including 1650 IBS patients, suggesting that using probiotics in IBS patients is better for symptom improvement, although there was heterogeneity between each study. In this study, the administration of probiotics was better than placebo in improving overall symptoms of IBS, and there was no difference between the various probiotics used, such as *Lactobacillus*, *Bifidobacterium*, and *Streptococcus* [44]. Several IBS studies have reported a positive effect of probiotics and a superiority over placebo, with some consensus on the effectiveness of IBS treatment in probiotics. However, due to controversies about IBS pathology, patient heterogeneity, and over the reproducibility and clarity for gut microbiota in IBS patients, additional RCTs are needed [45].

After finding the sex-difference in the WAS-induced microinflammation in 2016 [13] sex-difference to probiotics treatment was investigated in the present study. There are also some differences. In the present study, administration of probiotics by gavage was performed before induction of WAS, and increased stress due to gavage may have affected the results of the experiment. We observed visceral analgesia only in the female WAS group, which was different from our previous study which has shown visceral analgesia at in both males and females. This difference might be originated from the stress of the gavage before WAS suggesting that the females are more resilient to stress than males. Another point is that there was a difference in the FPO between male and female only after body weight adjustment in the previous study [13]. However, there was significant difference of FPO between male and female in the present experiment, which suggests more stress has been added owing to gavage before WAS in the present study similar to the visceral analgesia only in the female WAS group.

There are several limitations to this study. First, this study did not consider the estrous cycle of female rats. In female rats, the estrous cycle may affect stress test results, because threshold for stress may differ depending on the amount of estrogen secreted. However, Larauche et al. [18], have shown that there is no relationship between VMR to CRD and estrous cycle stage. Second, the trend of analgesia in the WAS + probiotics group was found but without statistical significance. This may be because the sample size is small to observe a significant effect, or the probiotics prevent the effects of WAS (especially through the inhibition of inflammation in females), such that WAS-induced analgesia does not appear. Third, as mentioned above, administration of probiotics using oral gavage may be a stress-inducing factor. However, we also gave normal saline to the control group. Psychological and emotional stress in daily life seems to have a role in symptom generation and exacerbation in patient with IBS. In addition, unresolved and repetitive symptoms without underlying etiology in patient with IBS also could be a cause of stress, resulting in a vicious cycle. This experiment using WAS in rats was conducted to investigate how stress affects gastrointestinal symptoms and immunity, and how probiotics affect these changes. Although this kind of experiment could not exactly reflect every pathophysiology of all types of IBS patients, the results of this experiment help us to understand the role of stress in GI physiology and could be applied to stress-related IBS patients. In addition, IBS patients showed a strong sex-related effect in terms of sociocultural relationships. However, in animal experiments, it might be difficult to explain this gender effect; instead it only reflects the sex differences. Thus, the WAS model may not be adequate for the explanation of gender predominance of IBS or its relation to the early and late onset of IBS.

In conclusion, chronic probiotic administration significantly reduced colonic mucosal mast cell count and had an anti-inflammatory effect in female rats. These results may help in understanding the role of probiotics in the reduction of IBS, and suggest the possibility of using mast cells and cytokines as therapeutic targets in female IBS patients.
